# Postoperative Left Anterior Descending Artery Compression and Left Ventricular Thrombus Following the Repair of a Penetrating Right Ventricular Injury in an Adolescent: A Case Report

**DOI:** 10.7759/cureus.98040

**Published:** 2025-11-28

**Authors:** Rory O'Donnell, Jacqueline Chung, Pranesh Jogia

**Affiliations:** 1 Cardiology, Waikato Hospital, Hamilton, NZL; 2 Critical Care Medicine, Waikato Hospital, Hamilton, NZL

**Keywords:** case report, echocardiography, left anterior descending artery occlusion, left ventricular thrombus, penetrating cardiac trauma, right ventricular injury

## Abstract

Penetrating cardiac trauma in adolescents is rare. Perioperative myocardial infarction secondary to surgical repair with subsequent left ventricular (LV) thrombus is even more so. A 16-year-old male sustained a stab wound to the left anterior chest. He collapsed and was brought to the emergency department by the family in a peri-arrest state. Emergency sternotomy revealed a right ventricular (RV) free wall laceration adjacent to the left anterior descending artery (LAD), repaired with pledgeted sutures. The LAD appeared patent intraoperatively. Postoperatively, ECG demonstrated new ST elevation, and transoesophageal echocardiography demonstrated new regional wall motion abnormalities. Coronary angiography showed mid-LAD occlusion, considered secondary to extraluminal compression from local postoperative tissue swelling and/or pledget tension. Stenting was not pursued, as extrinsic compression is unlikely to be relieved by percutaneous coronary intervention, and reoperation was considered high risk immediately post-sternotomy. Transthoracic echocardiography later revealed severe LV systolic dysfunction (ejection fraction of 29%) and a 1.5 × 1.0 cm apical thrombus. Anticoagulation with enoxaparin was initiated and transitioned to warfarin, together with guideline-directed heart failure therapy. The patient recovered and was discharged on day 18 after admission. Follow-up echocardiography was planned at three months to reassess function and thrombus resolution. This case illustrates survival following penetrating RV injury complicated by perioperative LAD occlusion and subsequent ischemic LV dysfunction with thrombus, highlighting anticoagulation and heart failure management challenges in an adolescent.

## Introduction

Penetrating cardiac trauma is a rare but highly lethal injury, with mortality frequently exceeding 50% even in modern trauma centers [1-4]. Most patients succumb before reaching hospital care, and survival is particularly poor when coronary arteries are involved or when left ventricular (LV) injuries occur [1,5]. Most published series involve adults, with pediatric and adolescent cases representing a small minority. In one large pediatric series, penetrating cardiac trauma accounted for <1% of trauma admissions, and in‑hospital survival was <30% [4]. Stab wounds are the main mechanism in civilian settings, and the right ventricle is most commonly injured because of its anterior position [4,5]. While LV thrombus is a recognized complication of large anterior myocardial infarction, it is infrequently reported in adolescents, and published descriptions in the context of perioperative coronary compromise after penetrating cardiac repair are limited. This case demonstrates LV thrombus formation secondary to mid-left anterior descending (LAD) artery occlusion after repair of penetrating cardiac injury, emphasizing the potential for delayed coronary compromise due to extraluminal compression at the repair site.

## Case presentation

A 16‑year‑old previously healthy male sustained a stab wound to the left anterior chest. He collapsed and was brought to the emergency department by the family in a shocked, peri-arrest state. The stab wound was explored, and it appeared evident that he had a laceration of the fourth to fifth ribs, and the stab had penetrated the chest. Bedside transthoracic echocardiography (TTE) demonstrated a large pericardial effusion; no further findings were noted, but the clinical picture was consistent with cardiac tamponade requiring immediate operative intervention. He was intubated, and a massive transfusion protocol was activated. Emergency thoracotomy converted to median sternotomy revealed a right ventricular (RV) free wall laceration adjacent to the LAD, a left internal thoracic vein injury (which was subsequently repaired), and probable left internal thoracic artery injury (which was ligated). According to the operative note, the RV laceration lay just medial to the LAD, and the artery was noted to be “very close and at risk of iatrogenic closure with sutures.” Two pledgeted sutures were placed to control bleeding, and the repair was reinforced with BioGlue in this region. There appeared to be flow through the LAD, and the left ventricle contracted well before closure. In total, he received seven units of packed red cells, two units of fresh frozen plasma, six units of cryoprecipitate, and tranexamic acid.

**Figure 1:**
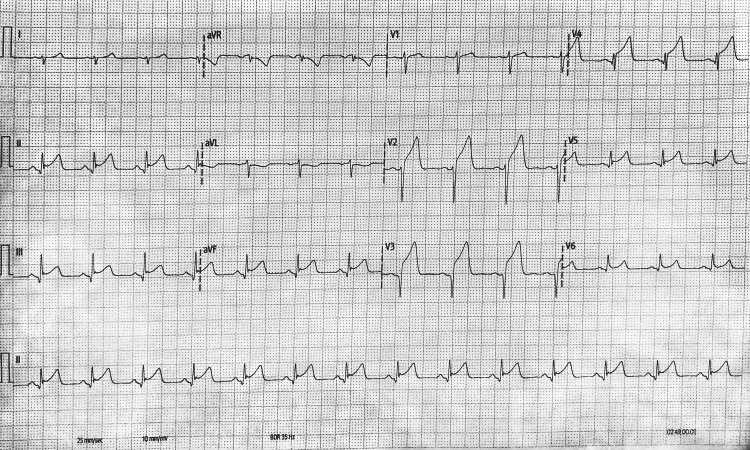
Electrocardiogram demonstrating ST-segment elevation in leads II, III, aVF, and V2-V6. The changes were noted in the postoperative state. The pattern raised the possibility of ischemia related to a wrap-around left anterior descending artery supplying both the anterior and inferior territories.

Given his significant blood loss, prolonged period of hemodynamic instability, and apparently reasonable intraoperative recovery, he was transferred directly to the intensive care unit (ICU) on low-dose noradrenaline. A 12-lead ECG was performed shortly after arrival to the ICU, approximately two hours after surgery, and showed new ST-segment elevation in leads II, III, aVF, and V2-V6 (Figure 1). No preoperative ECG was available due to the emergent presentation. The pattern raised the possibility of ischemia related to a wrap-around LAD supplying both anterior and inferior territories. Transesophageal echocardiography (TOE) subsequently demonstrated new septal and inferior akinesis, with RV lateral wall akinesia, prompting urgent coronary angiography.

**Figure 2:**
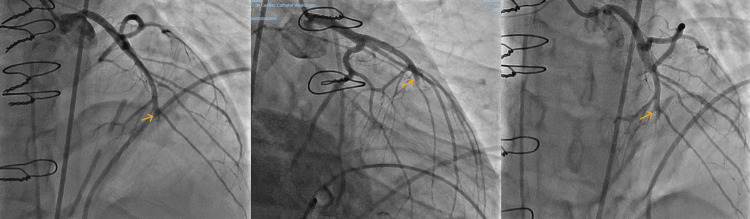
Coronary angiography in multiple projections of the left coronary system. Yellow arrows mark the site of mid-left anterior descending artery occlusion distal to the septal and diagonal branches.

Coronary angiography confirmed complete occlusion of the mid-LAD distal to the major septal and diagonal branches, with the left main, circumflex, and right coronary arteries free of disease (Figure 2). The vessel gave rise to several lateral branches and a large septal branch proximal to the point of occlusion. Following multidisciplinary review, the occlusion was attributed to external compression at the site of the RV repair, where the LAD lay immediately adjacent to the pledgeted sutures. Postoperative edema, hematoma, pledget tension, and possible BioGlue mass effect were considered likely contributors to this extraluminal compression.

After determining that the most likely cause of occlusion was extraluminal compression, further instrumentation, such as passing a wire, intravascular imaging, or attempting balloon angioplasty, was discussed but not undertaken due to the risk of disrupting the recent repair and worsening bleeding. In addition, the occluded segment lay beyond the most proximal LAD branches, and the patient remained hemodynamically stable following resuscitation. Immediate re-operation was deemed high risk, given re-entry after a traumatic RV repair risks disruption of the repair, loss of hemostasis, and uncontrollable bleeding in a patient recovering from hemorrhagic shock. A multidisciplinary decision was therefore made to proceed with medical management and close hemodynamic and imaging surveillance in the ICU.

Formal TTE on day one showed an ejection fraction (EF) of ~40%, with septal and inferior dyskinesis, distal apical akinesis, and severe RV dysfunction (Video 1). He was extubated within 24 hours and transferred to an enhanced recovery unit. Over the following week, he remained hemodynamically stable. Chest drains were removed on day eight, and analgesia was transitioned from patient‑controlled to oral agents. By day seven, repeat TTE demonstrated further deterioration in LV function (EF of 29%) and a 1.5 × 1.0 cm apical thrombus with spontaneous echo contrast (Videos 2, 3). Therapeutic anticoagulation was initiated with enoxaparin and transitioned to warfarin, with a target international normalized ratio (INR) of 2.0-3.0. The planned duration was approximately three months, with a repeat TTE in three months to assess recovery of EF and resolution of thrombus and to guide cessation of anticoagulant therapy. Guideline‑directed therapy for heart failure was commenced using low-dose initiation and gradual titration: sacubitril/valsartan (24 mg/26 mg twice daily) was commenced, together with bisoprolol (2.5 mg), spironolactone (25 mg), and empagliflozin (25 mg). Renal function, blood pressure, and serum potassium levels were monitored throughout the titration. The patient tolerated the therapy well and remained hemodynamically stable.

**Video 1:** Transthoracic echocardiogram (intensive care unit study) showing moderately reduced left ventricular systolic function (ejection fraction of ~40%) with dyskinesis of the mid-to-apical septal and inferior walls, and akinesis of the distal apex and mid-to-apical anterior wall.

**Video 2:** Transthoracic echocardiography demonstrating spontaneous echo contrast within the left ventricular apex and a 1.5 × 1.0 cm apical thrombus adherent to an akinetic apex, with additional regional wall motion abnormalities in the septal and inferior segments. Severe left ventricular systolic dysfunction is present (Simpson’s biplane ejection fraction of 29%).

**Video 3:** Transthoracic echocardiographic images of the left ventricular apex shown side by side with and without colour Doppler. The apical thrombus is delineated within an akinetic apex, with color Doppler confirming absence of flow.

During the second week, he mobilized independently and received education regarding anticoagulation and heart failure therapy. On day 18, he was discharged in sinus rhythm, hemodynamically stable, with wounds well healed and independence in activities of daily living. Discharge planning included review with his general practitioner for INR monitoring, cardiology follow‑up with repeat TTE at three months, and heart failure nurse review. He was advised to avoid lifting >2 kg and avoid driving for six weeks.

## Discussion

Penetrating cardiac trauma mandates rapid diagnosis and definitive operative management. Advanced Trauma Life Support principles emphasize airway and breathing stabilization, hemorrhage control, ECG and troponin assessment, and bedside TTE as initial steps. Operative repair remains the cornerstone for penetrating cardiac injuries; mortality remains high despite modern trauma systems [6-8].

In our patient, the RV defect was repaired with two pledgeted sutures placed to control RV bleeding immediately beside the mid-LAD, a configuration the operating surgeon specifically noted as carrying a risk of iatrogenic obstruction. Intraoperative assessment suggested reassuring LAD flow and satisfactory global ventricular contraction; however, this close spatial relationship still created the potential for delayed extraluminal compromise once postoperative edema, a small hematoma, BioGlue mass effect, or pledget tension developed, an anatomical scenario recognized in prior reports of suture-related coronary compromise [9,10]. The subsequent onset of new postoperative ST-segment elevation prompted urgent angiography, which confirmed mid-LAD occlusion at the level of the repair. This sequence highlights the importance of prompt coronary assessment when new ECG changes occur after cardiac repair [7]. It should also be noted that the TOE performed in the early postoperative setting was a focused scan in the ICU used to establish any regional wall motion abnormality rather than to establish a cause of extraluminal compromise.

Percutaneous coronary intervention was not undertaken because the obstruction was considered extraluminal. Angiography demonstrated a smooth, tapering occlusion without an intraluminal filling defect, making thrombus less likely and supporting an external compressive mechanism. Passing a wire or attempting balloon angioplasty was discussed; however, instrumentation risked disrupting the recent RV repair and provoking hemorrhage, and stenting would not have relieved an external compressive mechanism. In addition, the occluded segment lay distal to the major septal and diagonal branches, limiting its prognostic impact, and the patient remained hemodynamically stable. Urgent re-operation was also judged high risk in the early post-sternotomy period following hemorrhagic shock. A multidisciplinary decision was therefore made to pursue medical management with close hemodynamic and imaging surveillance in the ICU. CT coronary angiography was not pursued, as it would not have been feasible or diagnostically reliable in the immediate post-sternotomy period, given the need for heart-rate control and the presence of postoperative mediastinal air and hematoma. The immediate priority was initiation and optimization of guideline-directed heart failure therapy and anticoagulation. Serial TTE was planned to assess the recovery of LV function and the resolution of the apical thrombus. Should LV function fail to improve or regional wall motion abnormalities persist, cardiac MRI may be considered to evaluate myocardial viability. If viable myocardium is demonstrated, repeat coronary angiography and consideration of delayed revascularization could then be undertaken.

The mid‑LAD occlusion resulted in extensive septal and inferior akinesis, with subsequent development of an LV apical thrombus. This represents a recognized complication of perioperative anterior myocardial infarction, rather than a direct consequence of penetrating trauma itself. The 2022 American Heart Association (AHA) scientific statement recommends oral anticoagulation for approximately three months for established LV thrombus and advises against prophylactic anticoagulation in reperfused myocardial infarction without thrombus [11]. Vitamin K antagonists remain the standard therapy, and direct oral anticoagulants are considered reasonable alternatives, but supporting data is limited [11,12]. Warfarin was selected after initial low‑molecular‑weight heparin because evidence for direct oral anticoagulants in pediatric or trauma settings is sparse, and warfarin offers readily reversible anticoagulation. Follow-up TTE was planned at three months to guide the duration of therapy.

The 2021 European Society of Cardiology (ESC) guidelines for the diagnosis and treatment of acute and chronic heart failure advocate a regimen including an angiotensin-converting enzyme inhibitor or angiotensin receptor neprilysin inhibitor, a beta-blocker, a mineralocorticoid receptor antagonist, and a sodium-glucose co-transporter-2 (SGLT2) inhibitor to reduce mortality and hospitalization [13]. These recommendations are derived from adult clinical trials; evidence to guide pharmacologic therapy in children and adolescents is limited, and current practice largely involves extrapolation from adult data [14]. In this case, as the patient was physiologically adult in size and hemodynamics, guideline-directed therapy was initiated with gradual uptitration and monitoring of renal function, blood pressure, and serum potassium. The use of sacubitril/valsartan and SGLT2 inhibition in adolescents is off-label, and this was undertaken within a multidisciplinary team framework with informed discussion regarding the evidence gaps. Pediatric-specific data remain scarce, highlighting the need for prospective studies and structured follow-up.

No ventricular arrhythmias occurred during admission, and the LV dysfunction was considered potentially reversible. In accordance with ESC and AHA secondary-prevention guidance, implantable cardioverter-defibrillator implantation was not indicated, with deferred re-evaluation planned after recovery and optimization of medical therapy [15,16].

## Conclusions

This case demonstrates that delayed coronary compromise can occur even when coronary flow appears preserved at the time of repair for penetrating cardiac injury. New postoperative ST-segment elevation or regional wall motion abnormalities should prompt urgent coronary assessment to distinguish intraluminal occlusion from extraluminal compression related to surgical repair. The resulting perioperative myocardial infarction in this patient led to progressive LV dysfunction and subsequent apical thrombus formation, reinforcing the importance of serial imaging in the days following injury. Anticoagulation should follow guideline-based recommendations for established LV thrombus, and guideline-directed medical therapy for heart failure can be implemented in physiologically adult adolescents with careful monitoring. This case highlights the need for multidisciplinary decision-making, structured follow-up, and vigilance for evolving coronary and ventricular complications after penetrating cardiac trauma.

## References

[1] McNicoll CF, McNickle AG, Vanderet D (2023). Shot through the heart: a 17-year analysis of pre-hospital and hospital deaths from penetrating cardiac injuries. Injury.

[2] Parreira JG, Coimbra R (2025). Penetrating cardiac injuries: what you need to know. J Trauma Acute Care Surg.

[3] Isaza-Restrepo A, Donoso-Samper A, Benitez E (2023). Retrospective analysis of 261 autopsies of penetrating cardiac injuries with emphasis on sociodemographic factors. Sci Rep.

[4] Lustenberger T, Talving P, Lam L, Inaba K, Mohseni S, Smith JA, Demetriades D (2013). Penetrating cardiac trauma in adolescents: a rare injury with excessive mortality. J Pediatr Surg.

[5] Tang AL, Inaba K, Branco BC (2011). Postdischarge complications after penetrating cardiac injury: a survivable injury with a high postdischarge complication rate. Arch Surg.

[6] Gupta B, Singh Y, Bagaria D, Nagarajappa A (2023). Comprehensive management of the patient with traumatic cardiac injury. Anesth Analg.

[7] Ball CG, Lee A, Kaminsky M, Hameed SM (2022). Technical considerations in the management of penetrating cardiac injury. Can J Surg.

[8] Levine GN, McEvoy JW, Fang JC (2022). Management of patients at risk for and with left ventricular thrombus: a scientific statement from the American Heart Association. Circulation.

[9] Seltmann M, Achenbach S, Muschiol G, Feyrer R (2010). Suture-induced right coronary artery stenosis. J Cardiovasc Comput Tomogr.

[10] Urabe D, Ide M, Matsuoka M, Miyake R (2022). Iatrogenic right coronary artery occlusion during minimally invasive cardiac surgery-tricuspid annuloplasty-a case report. JA Clin Rep.

[11] Gaudino M, Dangas GD, Angiolillo DJ (2023). Considerations on the management of acute postoperative ischemia after cardiac surgery: a scientific statement from the American Heart Association. Circulation.

[12] Zhang Q, Zheng H, Zhang Z, Xu Y, Zhang W (2025). Advancing clinical management of left ventricular thrombosis: prevention, detection and treatment modalities in the modern era. Heart.

[13] McDonagh TA, Metra M, Adamo M (2021). 2021 ESC Guidelines for the diagnosis and treatment of acute and chronic heart failure. Eur Heart J.

[14] Ahmed H, VanderPluym C (2021). Medical management of pediatric heart failure. Cardiovasc Diagn Ther.

[15] Zeppenfeld K, Tfelt-Hansen J, de Riva M (2022). 2022 ESC Guidelines for the management of patients with ventricular arrhythmias and the prevention of sudden cardiac death. Eur Heart J.

[16] Al-Khatib SM, Stevenson WG, Ackerman MJ (2018). 2017 AHA/ACC/HRS Guideline for management of patients with ventricular arrhythmias and the prevention of sudden cardiac death: a report of the American College of Cardiology/American Heart Association Task Force on Clinical Practice Guidelines and the Heart Rhythm Society. Circulation.

